# Ciruvis: a web-based tool for rule networks and interaction detection using rule-based classifiers

**DOI:** 10.1186/1471-2105-15-139

**Published:** 2014-05-12

**Authors:** Susanne Bornelöv, Simon Marillet, Jan Komorowski

**Affiliations:** 1 Department of Cell and Molecular Biology, Science for Life Laboratory, Uppsala University, 751 24 Uppsala, Sweden; 2 Institute of Computer Science, Polish Academy of Sciences, 01-248 Warsaw, Poland; 3 Current address: INRIA Sophia-Antipolis-Méditerranée, Algorithms-Biology-Structure, Sophia-Antipolis, France

**Keywords:** Visualization, Rules, Interactions, Interaction detection, Classification, Rule-based classification

## Abstract

**Background:**

The use of classification algorithms is becoming increasingly important for the field of computational biology. However, not only the quality of the classification, but also its biological interpretation is important. This interpretation may be eased if interacting elements can be identified and visualized, something that requires appropriate tools and methods.

**Results:**

We developed a new approach to detecting interactions in complex systems based on classification. Using rule-based classifiers, we previously proposed a rule network visualization strategy that may be applied as a heuristic for finding interactions. We now complement this work with Ciruvis, a web-based tool for the construction of rule networks from classifiers made of IF-THEN rules. Simulated and biological data served as an illustration of how the tool may be used to visualize and interpret classifiers. Furthermore, we used the rule networks to identify feature interactions, compared them to alternative methods, and computationally validated the findings.

**Conclusions:**

Rule networks enable a fast method for model visualization and provide an exploratory heuristic to interaction detection. The tool is made freely available on the web and may thus be used to aid and improve rule-based classification.

## Background

Technological developments have increased the ability to generate and store large amounts of data. However, for the data to be useful relevant methods for their analysis are needed. Classification methods are algorithms that automatically learn from such large data sets; however, the requirements on such methods are quite high and the need for new classification methods have been stressed, especially the need for methods that are able to identify interactions in the data [1-3]. For instance, single nucleotide polymorphisms (SNPs) found in genome-wide association studies using traditional statistical analysis can only explain small fractions of many common diseases [4] and classifiers using those markers may be of poor quality [5]. It has been suggested that this is due to the lack of gene-gene and gene-environment interactions in the models [1] and efforts have been made to develop specific tools, e.g. for the identification of SNP interactions [6].

Rule-based classifiers are one type of classifiers. Their strength lies in the fact that they are comparably easy to interpret while still producing models of reasonable quality, which have made them suitable for applications in systems biology. Rule-based classifiers have earlier been applied to a wide spectrum of problems in genomics, proteomics, epigenetics, e.g., predict gene ontology terms from gene expression time profiles [7], to interpret microarray data [8], to model cleavage of polypeptide octamers by the HIV-1 protease [9], to model ligand-receptor interactions [10], and to classify Alzheimer’s patients [11].

A rule-based classifier consist of a set of IF-THEN rules that describes the relations in the training data almost in natural language based on the original feature names. There are different software packages that can generate rules including ROSETTA [12], and WEKA [13]. Rule-based classifiers are non-linear and the identified rules may describe important features and interactions in the data. An intuitive heuristic to identify putative interactions from a set of rules is to search the rules for combinations of conditions that occur frequently in them. However, a classifier typically contains a large number of rules, which sometimes may be very complex with five to ten, or even more, conditions. Thus, new tools are needed to support the visualization and interpretation of the rules.

Most attempts to visualize rules have concerned association rules. For an overview of such visualization techniques, see for example [14,15]. Software previously developed for this task includes the R package *arulesViz*[16] that uses a two-dimensional matrix in which similar rules are clustered. However, most methods scale poorly with an increased number of rules. We were impressed by the readability of the circular graphs produced by the Circos software [17] and decided to use it for rule visualization. To our knowledge, the only attempt to visualize rules in a circular layout was done for association rules by [18].

We therefore present *Ciruvis*: a web-based tool [19] for the visualization of conditions that are associated in the rules using a circular layout. It relies on a scoring system previously introduced by [20] for which we now provided a free-to-use web-based implementation. The tool may produce both separate rule networks for each decision outcome and a combined network. In this study we focused on the detection of interaction effects in those networks, although they may also be valuable solely for visualization purposes.

Using different types of simulated data sets, we showed that applying our tool to ROSETTA rules may identify interactions in the data. Furthermore, we applied the tool to real data in order to compare it to other methods and to illustrate its use. The tool is fast, scales well with the number of rules and is easy to use.

In conclusion, we believe that Ciruvis may facilitate visualization of rule-based classifiers and the discovery of interactions.

## Methods

### Rule terminology

A rule describes a relation between the rule conditions (the left-hand-side, LHS, of the rule) and the rule outcome (the right-hand-side, RHS). For example, a rule taken from a classifier for leukemia based on gene expression is: **IF** *MIF*=‘high’ **AND** *GPX1*=‘low’ **THEN** *type*=‘chronic lymphocytic leukemia’.

The rule *support* is the number of objects that fulfill the LHS of the rule, and the *accuracy* is the fraction of those objects that also fulfill the RHS of the rule, or equivalently, *accuracy* = P (RHS|LHS). A rule condition has the form *feature*=‘value’ (for example *MIF*=‘high’) and a rule may have one or multiple conditions. The rule outcome has the form of *class*=‘value’ and there is only one such feature.

### Definition of the rule network

Ciruvis is a tool to visualize combinations of rule conditions that are important for a particular rule outcome. Each condition that has at least one connection to another condition is placed as a node on the outer ring of the circle in an alphabetical order. Two conditions are connected inside the circle if they co-occur in some rule (s). The score of the connection between two conditions, *x* and *y*, is defined as

$$\text{connection}\left(x,y\right)=\sum_{r\in R\left(x,y\right)}\text{support}\left(r\right)\cdot \text{accuracy}\left(r\right)$$

where R*(x*,*y*) is the set of all rules in which *x* and *y* co-occur.

The connections are shown as edges between the nodes. The width and color of the edges are related to the connection score (low = yellow and thin, high = red and thick). The inner ring shows the color of the condition on the other side of the connection. The width of a node is the sum of all connection to it, scaled so that all nodes together cover the whole circle.

### Parameters and user interface

To run Ciruvis, a rule file must be submitted either in the ROSETTA or in a line-by-line format. Several optional filtering and formatting parameters are available (Additional file 1: Table S1). A screen shot from the submission form and the results page are shown in (Additional file 2: Figure S1). One rule network is generated for each possible outcome. The figures are interactive, and by clicking on the edge between two conditions, all rules containing that combination of conditions are shown. If the Ctrl key is held while selecting multiple edges, the intersections of rules from these edges are shown. The name of a node is shown when the mouse is hovered over it. It is possible to download the Ciruvis figure in the Scalable Vector Graphics (SVG) format and the feature labels as an HTML table which both can be easily edited and used to produce publication-quality figures.

### Generation of simulated data

We used simulated data to test the ability to detect interactions using the networks. The dataset was constructed to contain both noise, features correlated to the decision, and pairs of interacting features. The interacting features were defined so that they together were predictive for the decision but that each of them was uncorrelated to it. Translated into a real-world situation, this could represent a situation with SNPs of which some lack marginal effects on the outcome, but have an interaction effect caused by gene-gene interactions or epistasis.

For each data set we defined five correlated features with expected correlation *c* = X*i/4, where *X* was the maximal correlation for that data set and *i* = 0, 1, …, 4. Each correlated variable was named after the index *i* and its correlation *c* according to C*i*_*c*. Similarly, we defined five pairs of interacting features which, when taken together, were predictive for the outcome with the probability *p* = Y*i/4, where *Y* was the maximal value for that data set and *i* = 0, 1, …, 4. The features of the pairs were named R*i*_ *p* and S*i*_ *p* where *i* was an index 0 < = *i* < = 4, and *p* was their probability of being predictive.

Each choice of the parameters *X* and *Y* thereby represented one data set with 15 features. In order to generate datasets with different properties, we allowed *X* and *Y* to take all values in {0.00, 0.05, 0.10, …, 0.95, 1.00}, which defined 21*21 = 441 datasets. In each dataset 1000 objects were generated using the algorithm below. Note that the *Random()* function returns only discrete values and thus, that both the decision and the features are discrete.

Here *Random*() is a function that returns 0 or 1 with equal probability, and *Probability*(*q*) is a function that returns *true* with probability *q* and *false* otherwise. We generated 50 replicate data sets for each combination of *X* and *Y* and trained a classification model on each of those. The classification accuracies presented were the averaged over those 50 models and all rules from the replicates were merged together for Ciruvis to construct an average picture.

### Rule-based classification using ROSETTA

The rule-based classifiers were constructed using the ROSETTA toolkit for analysis of tabular data [12,21]. ROSETTA is a mathematical framework capable of deriving IF-THEN rules from a set of training examples. Boolean reasoning is used to compute minimal sets of features, called reducts, able to discriminate between the training examples equally well using all features. Based on the feature values in the training data, the reducts are transformed into rules that describe minimal sets of feature conditions associated with a particular decision class. Combined, these rules may be used to classify previously unseen objects.

Algorithms and parameters are described shortly in the results section and in more detail in the Supplementary methods (Additional file 3: Supplementary methods). The quality of each classifier was measured by the classifier accuracy (the proportion of correctly classified objects) which was estimated using 10-fold or leave-one-out cross validation.

## Results and discussion

### Detection of correlated versus interacting features in simulated data

To investigate how well the rule networks from Ciruvis could detect feature interactions, we first tested it using simulated data. The data contained both features correlated to the decision and pairs of interacting features predictive for the decision. The level of correlation and pairwise predictability was determined by two parameters that defined a maximum level for the most predictive feature/pair in the dataset. The maximum level of correlation, *X*, and of interaction, *Y*, was varied between 0 and 1. Then, for each data set the number of correctly classified objects was counted (Additional file 4: Figure S2). As expected, there were usually more correctly classified objects when the features were more predictive (as measured by higher *X* and/or *Y*). Surprisingly, a higher level of interaction increased or at least retained the classification quality, whereas a higher correlation sometimes decreased the quality. Specifically, the quality was decreased when the pairwise correlation was high and the correlation increased over 0.20-0.30. When the interaction level was 1.00 this was the most evident, since the average number of correctly classified objects decreased from 998–999 out of 1000 for *X* < 0.25 to a local minimum 828 at *X* = 0.45.

This suggests that the rule generation algorithm was biased towards finding rules containing features correlated to the decision. When the correlated features were not present, then the combinatorial rules of higher quality were more likely to be found. The identified masking became one of the focuses in our study.

**Figure 1:**
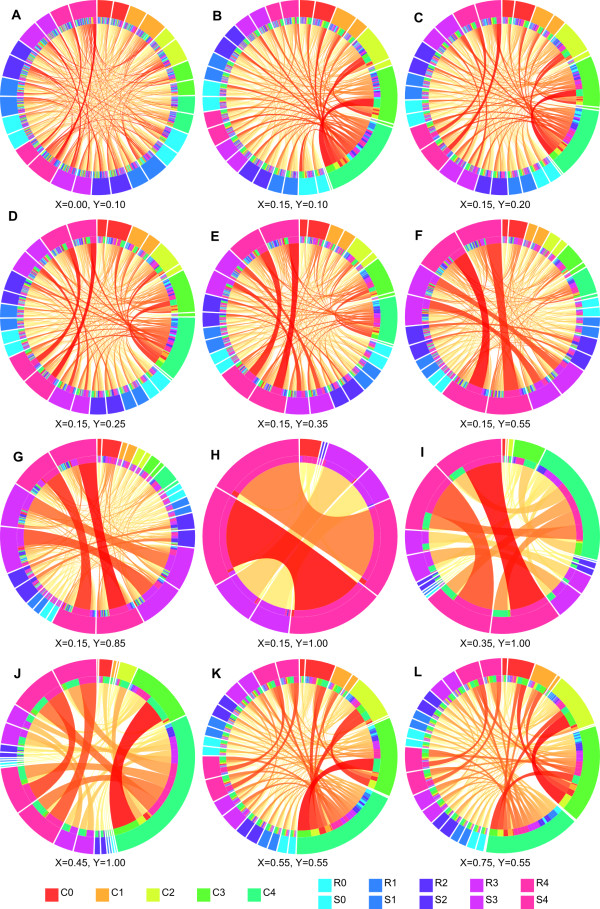
**Rule networks for simulated data.** Rule networks for twelve different pairs of maximum correlation *X* and interaction *Y* for the “0” outcome. The parameter choices **(A-L)** correspond to points in Additional file 4: Figure S2. The correlated features are named C0 to C4 (lowest to highest correlation), and the pairs R0, S0 to R4, S4 (lowest to highest correlation). The colors were specified so that the interacting pairs have the same color. Each feature occurs twice in the figure; the first time with the value 0 and the second with 1.

Next, we investigated the behavior of the rule networks for different datasets (Figure 1). Since both the features and the decision were binary only the networks for outcome “0” are presented. Based on the data generation algorithm opposite values of the R and S variables were expected to predict the “0” decision, e.g., **IF** R = 0 **AND** S = 1 **THEN** DEC = 0, whereas equal values predict the “1” decision. The aim was to observe how small interactions could still be detected and to learn about their properties; for instance, whether they would be masked by features strongly correlated to the decision.

Using *X* = 0.00 and *Y* = 0.10 we could identify visible connections between pairs with an interaction level at 10, 8, and 5% (Figure 1A). The connections between “R4_10” and “S4_10” were the two strongest in the figure demonstrating that very weak interactions may be detected in the Ciruvis networks even in the presence of very noisy data. This particular example also illustrated that the rules from a classifier may be informative, even when the quality of classification is essentially not better than “random guessing”.

In the following runs we processed datasets with a small background correlation, *X* = 0.15 (Figure 1B-H). With *Y* = 0.10 the pair with a 10% chance of interaction was barely visible, and not among the highest scored connections in the figure (Figure 1B). As *Y* was increased the two (or three) highest scored pairs became step-by-step more visible (Figure 1C-E) and when *Y* was set to 0.55 or higher the three most interacting pairs were by far the strongest connections (Figure 1F-G), with the exception of *Y* = 1.00 when the third pair (R2 + S2) was masked by the more predictive pairs (Figure 1H).

Similarly, when the best interaction was 100% predictive (*Y* = 1.00) and with higher correlation (*X* = 0.35 or *X* = 0.45, respectively), the strongest interacting pair was highly visible and the second pair had indeed a visible connection, but it was on the same level as some of the noise (Figure 1I-J). Although it is useful to know that stronger rules may mask weaker ones, masking caused by perfect correlation would normally not be expected in a real data set.

When the dataset had both a high level of correlation and interactions, the connections for the two strongest interacting pairs were visible, but not the strongest connections (Figure 1K-L). However, the true interactions are shown as connections from conditions with otherwise few and weak connections, while connections that are artifacts caused by combinations of correlated features origin from conditions with a lot of strong connections.

An observation in all of the generated rule networks was that at most three (out of four non-zero) interacting pairs appeared in the networks. A likely explanation is that the stronger interactions mask the weaker ones, similarly to how strong correlations do.

### Removal of correlated features decreased the masking of weak interactions

**Figure 2:**
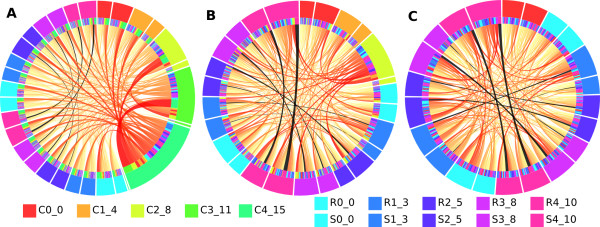
**Correlated features mask weak interactions.** Rule networks for the outcome “0” in the simulated data. The data parameters are *X* = 0.10, *Y* = 0.15. **(A)** Using all features, **(B)** after the removal of the two strongest correlated features C4_15 and C3_11 and, **(C)** after the removal of the four strongest correlated features C1_4-C4_15. Connections between interacting features were colored black.

In the previous section we showed that when features correlated to the decision were roughly as strong or stronger than the interacting pairs, the latter were masked by the former. Subsequently, rules containing the interacting pairs were rarely found or barely visible in the rule networks. To investigate whether the removal of correlated features from the data would benefit to the detection of the pairs, we used the data from Figure 1B (in which the pairs are heavily masked) and removed the correlated features C4 and C3 (15% and 11% correlation, respectively). The pair with the highest interaction (R4 + S4, with interaction frequency 10%) subsequently became relatively stronger (Figure 2A-B). For instance, in Figure 2A the connection score between “S4 = 1” and “R4 = 0” is 0.7% of the total score in the figure, which increases to 1.8% in Figure 2B; becoming the strongest connection in the figure. The increase for the combination “S4 = 0” and “R4 = 1” was smaller but still significant, from 0.6% to 1.1%. In addition in Figure 2B the “R3 = 0” and “S3 = 1” pair could be identified (increased from 0.4% to 0.7%), although the connection was still weak. When the last two correlated features (C2 and C1 with 8% and 4% correlation, respectively) were removed as well, the strength of the first and the second pair increased sharply (to 4.3% and 1.4% respectively) (Figure 2C).

### Comparison to other methods using real data

In order to compare the interaction detection to other methods, and to apply the methodology to real data, we used the *California Housing*[22] dataset downloaded from [23]. This dataset was chosen as it had previously been subject for interactions detection [24].

*California Housing* describes housing value based on 1990 census data in California. The decision is the median value of a block group (*medianHouseValue*) and there are 8 features. We discretized the decision into three groups; one group of houses valued ≥500 000 which was encoded as ‘2’ , the remaining houses were split at their median into the intervals 0–173 600 and 173 601–499 999 (encoded as ‘0’ and ‘1’ , respectively). We used the features *longitude*, *latitude*, *housingMedianAge*, *total Rooms*, *population*, and *median Income* previously selected by [24] to build a rule-based model using ROSETTA. The numeric features were discretized using *EqualFrequencyBinning* with 4 intervals. The model accuracy was estimated using 10-fold cross validation.

**Figure 3:**
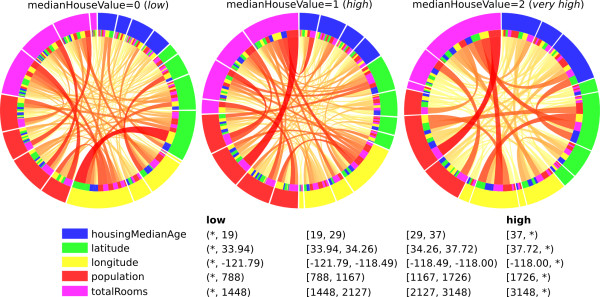
**Rule networks for regression data.** Rule networks for the California housing data after removal of the *medianIncome* feature. The features are indicated by node color, and the condition values are shown in increasing order (low, middle-low, middle-high, high) on the circle.

The *medianIncome* feature was highly correlated to the decision (r = 0.61; Additional file 5: Figure S3) and when the rule-based model was built to include it, it dominated the strongest connections (Additional file 6: Figure S4). An alternative model was built excluding *medianIncome* which reduced the accuracy of the model from 72.4% to 66.5% as important information was excluded, but made the identification of interacting pairs easier. Inspecting the rule networks (Figure 3), we identified the ten strongest connections for each outcome (Additional file 7: Table S2). For instance, for *medianHouseValue* = 0 three of these described combinations of conditions with specific values for *latitude* and *longitude*, three combinations with *population* and *totalRooms*, two with *population* and *longitude*, and two with *totalRooms* and *longitude*. For each one of these specific combinations of features, we computed whether it had a significant interaction effect (see Additional file 3: Supplementary methods for details). Additionally, we computed the expected accuracy (Additional file 7: Table S2) by first estimating the effect of each condition separately and then combining these effects under a multiplicative model (see [25] for a mathematical derivation). The interaction effects could then be assessed by comparing the observed and the expected accuracies.

Out of the ten strongest connections for *medianHouseValue* = 0 three were describing significant interactions. For instance, “*population* = [1167, 1726) AND *totalRooms* = [1448, 2127)” had an accuracy of 67.8% compared to an expected 51.7%. This increase in accuracy is due to a specific interaction between the population in the area and the total number of rooms. Supposedly, the number of rooms *per capita* is what determines the house prices.

In conclusion, an interaction between *population* and *totalRooms* was described by several connections. Additionally, a specific combination of *latitude* and *longitude* described an interaction predictive for low house prices, and a combination of high *houseMedianAge* and high *totalRooms* described an interaction predictive for very high house prices. Two of these pairs were reported as interacting by [24], but the third one is novel. The interaction between *latitude* and *longitude* was very strong in the previous study and it indeed appeared in several of the strongest connections. However, only one specific combination of conditions showed a significant interaction effect. This is most likely due to these two features being strongly correlated (r = -0.92; Additional file 5: Figure S3) and the assumption of independent effects therefore underestimated their interaction.

### Applications to leukemia and lymphoma

Finally, we applied Ciruvis to biological data describing leukemia [26] and lymphoma [27]. The leukemia set contained gene expression for 7129 genes from 38 patients divided into two different outcomes: acute lymphoblastic leukemia (ALL; *n* = 27) and acute myeloid leukemia (AML; *n* = 11). The lymphoma set contained 4026 genes from 62 patients divided into three outcomes: lymphoma and leukemia (DLCL or D; *n* = 42), follicular lymphoma (FL or F; *n* = 9) and chronic lymphocytic leukemia (CLL or C; *n* = 11). The probe names were changed into gene names when possible and otherwise kept as in the source data. A single quote was used to discern between multiple probes matching the same genes. Since most genes had their expression discretized into two intervals by ROSETTA (see Additional file 1: Supplementary methods for details on the discretization) the intervals were renamed into “low” and “high”, with the addition of “medium” if applicable. See (Additional file 8: Table S3 and Additional file 9: Table S4) for details on gene names and values.

**Figure 4:**
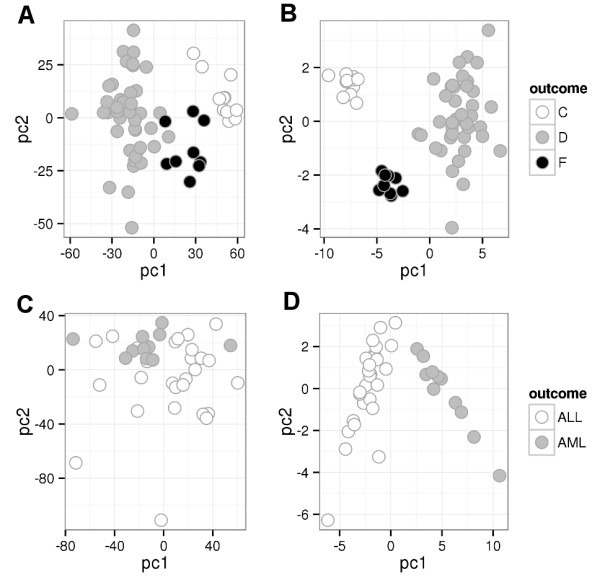
**Feature selection for leukemia and lymphoma.** The separation of the outcomes (disease types) using the first two principal components was improved when the 30 most significant features were used instead of all features. The figures show **(A)** lymphoma before, **(B)** lymphoma after, **(C)** leukemia before, and **(D)** leukemia after feature selection, respectively.

Firstly, we used Monte Carlo feature selection [28] to rank the genes by significance. After correcting for multiple testing, there were 701 significant (p < 0.05) genes for leukemia and 512 for lymphoma. Details about the feature selection are described in the Supplementary methods (Additional file 3: Supplementary methods). A principal component analysis (PCA) verified that using the 30 most significant features the outcomes were separable by the first two principal components (Figure 4). Missing values were replaced by the gene average during the PCA. Performing a disease association analysis using WebGestalt [29] we could confirm that the top ten disease associations of the selected genes contained annotations related to lymphoma and leukemia. For example the leukemia data were enriched for genes related to Lymphoid Leukemia (*LYN*, *CCND3*, *TCF3*, *CD33*, and *MYB*; adjP = 0.024) and the lymphoma for Acute Myeloid Leukemia (*CALR*, *SUMO*, and *MYB*; adjP = 0.18) and Acute Erythroblastic Leukemia (*PCBP2* and *MYB*; adjP = 0.18). The p-values were calculated by WebGestalt using the hypergeometric distribution and adjusted with Bonferroni correction.

Next, we used ROSETTA to train a rule-based classifier based on the selected features. The accuracy of the classifier was 100% for both data sets, estimated by leave-one-out cross validation. Details on the classification are described in the Supplementary methods (Additional file 3: Supplementary methods).

Since each rule set in the leave-one-out cross validation was trained from all objects except one, they are expected to be very similar to rules trained on the whole data. Therefore, instead of repeatedly training a classifier on the whole data, we merged all the rules from the cross validation iterations. Duplicates were removed and the rules were filtered so that rules that are supersets of other rules were removed if they had lower significance (hypergeometric distribution); for details on the p-value calculations, see [30]. The motivation behind the filtering strategy is that shorter rules are preferred if they are at least equally significant as their longer counterparts.

The filtered set of rules was submitted to Ciruvis using default parameters. The interactive rule networks are available online at [31].

**Figure 5:**
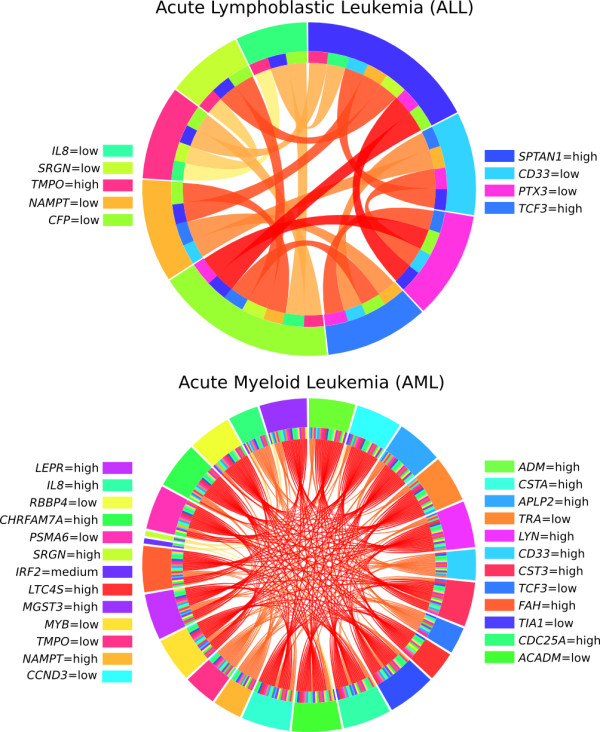
**Rule networks for leukemia.** Rule networks showing which rule conditions that are associated for leukemia. All connections are based on one rule each and are therefore of roughly equal score. The labels on each side of the figure are written the same order as the conditions appear in the figure.

The rule network for leukemia is shown in Figure 5. The difference in the overall topology of the networks for ALL and AML may partly be explained by a different number of rules for each outcome (48 for ALL and 254 for AML). Direct comparison between the networks was therefore difficult, since the same width would relate to a different number of rules. Instead we studied the strongest connections in each network. For this dataset both networks were quite simple, with all connections supported by only one high-quality rule. For ALL the highest scoring connections were based on any pair of the following conditions: *SPTAN1* = high, *PTX3* = low, and *CFP* = low; the conditions *SPTAN1* = high and *CFP* = low were the most frequent in other rules as well. Had the set of patients been larger, noiseless relationships would likely have been harder to identify and Ciruvis might have helped us identify the most important pairs out of more complicated rules. The AML network showed the same property, with a large number of connections based on only one rule with a pair of conditions. Most likely, the reason why more combinations were found in this network was that no single condition constituted a high quality rule in itself which forced the generation of longer rules.

**Figure 6:**
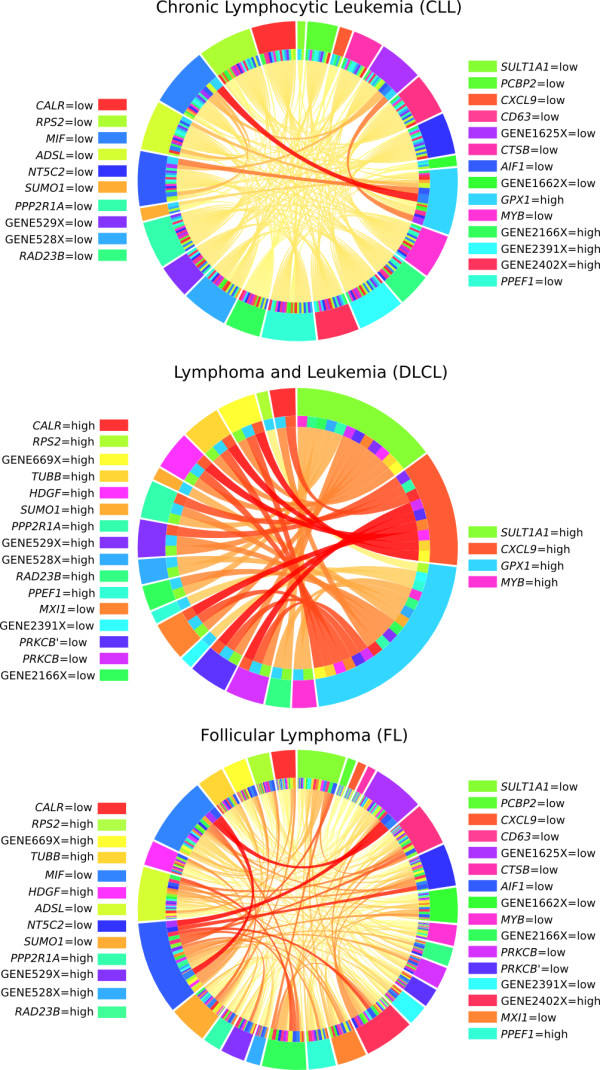
**Rule networks for lymphoma.** Rule networks showing which rule conditions that are associated for lymphoma. The labels on each side of the figure are written the same order as the conditions appear in the figure.

Similar behavior was observed in some of the rule networks for the lymphoma data (Figure 6). For CLL many connections were based on only one rule. The strongest connection (between *MIF* = low and *GPX1* = high) was based on four rules. This combination corresponded to a rule with 73% accuracy, compared to an expected accuracy of 51% assuming independent and multiplicative effects, which indicated that an interaction could be present. The second strongest connection was between *NT5C2* = low and *GPX1* = high which showed an accuracy of 84% compared to the expected 55%. A three-way interaction could be hypothesized and tested between *NT5C2* = low, *MIF* = low and *GPX1* = high with accuracy of 92% compared to the expected 83%.

The connections for the next outcome, DLCL, were supported only by one rule of high quality. Apparently, adding more conditions did not yield a significant increase in the rule quality. Notably, there are groups of conditions in the network that are interchangeable in certain rules. For instance, *CXCL9* = high may be combined with either of *PRKCB* = low, *PRKCB’* = low, *MXI1* = low, *HDGF* = high, *TUBB* = high and GENE669X = high to produce a rule for DLCL supported by all of the 42 patients in that group and with 100% accuracy. If instead *GPX1* = high is combined with any of the six genes the second highest scoring connections are achieved with rules that are almost as good; supported by 41 patients and with 100% accuracy.

For the FL outcome, a hypothesized three-way interaction between GENE1625X = low, *MIF* = low and *NT5C2* = low had to be rejected as the combined accuracy was lower than the predicted. Pairs of these conditions were separating FL + CLL from DLCL and together with any of several other conditions they defined three-way interactions.

## Conclusions

The requirements on classification methods to be user friendly and easy to interpret have increased over the past years. In that respect, rule-based classifiers which consist of IF-THEN “sentences” (or rules) make the models comparably easy to interpret. However, when the model has too many rules to be conveniently read, methods for visualization of the rules become important. We developed a web-based tool for rule visualization that is compatible with any type of classification rules. Its primary use is to provide a fast and easy visualization of a rule-based classifier. However, interpreting the rule networks can also help to generate hypotheses about feature interactions; which was the main focus of this study. A limitation of rule-based models is that the attributes have to be discrete, but discretization techniques help overcome this.

Using simulated data, we showed that the ROSETTA software may be used to construct rules that describe interactions even if the features lack marginal effects. Yet the rule detection may be biased towards features strongly correlated to the decision. We modeled different trade-offs between correlated and interacting features, and demonstrated to what degree stronger associations mask weaker ones.

The masking is a consequence of the classification algorithm, which is biased towards using the most predictive features for classification, omitting weaker but still predictive features or feature combinations. The problem arises when the interpretation of the classifier is important. To detect masking features, correlations between each feature and the decision may be computed or Ciruvis may be used to identify nodes with connections to almost all other nodes. We introduced a strategy in which the features most strongly correlated to the decision are removed from the data and the model is re-generated, in order for weaker interactions to gain importance for the classifier and the Ciruvis network.

An important difference as compared to other methods for interaction detection is that the rule networks are based on feature-value pairs (conditions) that tell us more precisely what feature values are involved in the interactions. Although not all the connections that were found in the networks were true interactions, the rule network is a fast method to generate a set of hypotheses to be further validated using other methods and new data.

In a comparison using data that have previously been used for interaction detection, we could identify both the reported interactions and a possibly novel one. Surprisingly, the strongest interaction previously reported (*longitude* and *latitude*) was found several times in the network, but appeared as significant only once. This interaction was based on two strongly correlated features that contradicted the assumption of independent effects.

Finally, we applied the tool to leukemia and lymphoma data. Our classification was very successful with 100% accuracy in the cross validation for both outcomes, similarly to what has been reported previously using multiple classification techniques [26,28]. The rule visualization provided a fast overview of the rule models and showed that there was very little overlap of conditions between the rules. This was likely caused by the small number of objects which allowed the individual rules to be of high quality; thus without the need for the rule-generation algorithm to construct a set of partly overlapping rules. Using the rule networks we were able to observe several possible interactions, of which many were computationally validated on our data. We believe it would be worth studying those interactions further and ultimately to validate them experimentally.

By making the Ciruvis freely available on the web [19] we hope that it will benefit the further research on rule-based classifiers and interactions. Additionally, since decision trees are commonly used and may be translated into rules, the application of the tool on decision trees would also provide an interesting extension.

## Competing interests

The authors declare that they have no competing interests.

## Authors’ contributions

JK and SB conceived the study. SB performed the experiments and analyzed the data. SM and SB implemented and developed the web-based tool. JK supervised the work. SB drafted the manuscript with input from JK and SM. All authors read and approved the final manuscript.

## Supplementary Material

Additional file 1: Table S1 Description of parameters and possible values for the rule submission form. Click here for file

Additional file 2: Figure S1 **(A)** Ciruvis submission form. **(B)** Ciruvis figure for the selected outcome “1” (high). Rules for the selected connection between *totalRooms* = [3148,*) and *medianIncome* = [4.7435,*) are shown to the right. Click here for file

Additional file 3 **Supplementary methods.** Supplementary description of the methods. Click here for file

Additional file 4: Figure S2 The number of correctly classified objects varied for different maximal correlation (*X*) and level of interaction (*Y*). The points A-L here represent the different parameters choices in Figure 1. The average standard error of the number of correctly classified objects in the replicates with the same X and Y was 12.2 (95% CI 0.0-22.5), with datasets with the lowest X and Y having the highest variation. Click here for file

Additional file 5: Figure S3 Correlation between pairs of features and decision in the California Housing dataset are displayed in the upper half as filled circles with size relative to the correlation and in the lower half as values. Positive correlations are colored from white to blue (highest) and negative correlations from white and red (highest). Click here for file

Additional file 6: Figure S4 Rule networks for the California housing data including the *medianIncome* feature. The color of the nodes shows which feature it is, and the condition values are shown in increasing order (low, middle-low, middle-high, high) on the circle. Click here for file

Additional file 7: Table S2 Calculation of relative risks (RR) and their confidence intervals (CI) for each of the ten strongest connections for each outcome, as well as the expected (exp) values. Connections that had a RR significantly greater than what would be expected assuming independent effects are marked with yellow background and may indicate interaction effects. An asterisk ‘*’ in the intervals denotes + or - ∞. Click here for file

Additional file 8: Table S3 The 30 most significant features for the lymphoma data (p-values calculated by MCFS). The original name refer to the internal name in the source data set. The gene name is given whenever it was available. The range for the discretized expression values are given as Low and High. Click here for file

Additional file 9: Table S4 The 30 most significant features for the leukemia data (p-values calculated by MCFS). The original name refer to the internal name in the source data set. The gene name is given whenever it was available. The range for the discretized expression values are given as Low, Medium (if applicable) and High. Click here for file

## References

[B1] Moore JH Asselbergs FW Williams SM Bioinformatics challenges for genome-wide association studies Bioinformatics 2010 26 4 445 455 10.1093/bioinformatics/btp713 20053841 PMC2820680

[B2] Schwarz DF Konig IR Ziegler A On safari to Random Jungle: a fast implementation of Random Forests for high-dimensional data Bioinformatics 2010 26 14 1752 1758 10.1093/bioinformatics/btq257 20505004 PMC2894507

[B3] Touw WG Bayjanov JR Overmars L Backus L Boekhorst J Wels M van Hijum SA Data mining in the Life Sciences with Random Forest: a walk in the park or lost in the jungle? Brief Bioinform 2013 14 3 315 326 10.1093/bib/bbs034 22786785 PMC3659301

[B4] Manolio TA Collins FS Cox NJ Goldstein DB Hindorff LA Hunter DJ McCarthy MI Ramos EM Cardon LR Chakravarti A Cho JH Guttmacher AE Kong A Kruglyak L Mardis E Rotimi CN Slatkin M Valle D Whittemore AS Boehnke M Clark AG Eichler EE Gibson G Haines JL Mackay TF McCarroll SA Visscher PM Finding the missing heritability of complex diseases Nature 2009 461 7265 747 753 10.1038/nature08494 19812666 PMC2831613

[B5] Jakobsdottir J Gorin MB Conley YP Ferrell RE Weeks DE Interpretation of genetic association studies: markers with replicated highly significant odds ratios may be poor classifiers PLoS Genet 2009 5 2 e1000337 10.1371/journal.pgen.1000337 19197355 PMC2629574

[B6] Wan X Yang C Yang Q Xue H Tang NL Yu W Predictive rule inference for epistatic interaction detection in genome-wide association studies Bioinformatics 2010 26 1 30 37 10.1093/bioinformatics/btp622 19880365

[B7] Lagreid A Hvidsten TR Midelfart H Komorowski J Sandvik AK Predicting gene ontology biological process from temporal gene expression patterns Genome Res 2003 13 5 965 979 10.1101/gr.1144503 12695321 PMC430886

[B8] Calvo-Dmgz D Gálvez JF Glez-Peña D Gómez-Meire S Fdez-Riverola F Using variable precision rough set for selection and classification of biological knowledge integrated in DNA gene expression J Integr Bioinform 2011 9 3 199 199 22829570 10.2390/biecoll-jib-2012-199

[B9] Kontijevskis A Wikberg JE Komorowski J Computational proteomics analysis of HIV-1 protease interactome Proteins 2007 68 1 305 312 10.1002/prot.21415 17427231

[B10] Strombergsson H Kryshtafovych A Prusis P Fidelis K Wikberg JE Komorowski J Hvidsten TR Generalized modeling of enzyme-ligand interactions using proteochemometrics and local protein substructures Proteins 2006 65 3 568 579 10.1002/prot.21163 16948162

[B11] Kruczyk M Zetterberg H Hansson O Rolstad S Minthon L Wallin A Blennow K Komorowski J Andersson MG Monte Carlo feature selection and rule-based models to predict Alzheimer’s disease in mild cognitive impairment J Neural Transm 2012 119 7 821 831 10.1007/s00702-012-0812-0 22573144

[B12] Komorowski J Øhrn A Skowron A Klösgen WZJ The ROSETTA Rough Set Software System Handbook of Data Mining and Knowledge 2002 New York: Oxford University Press

[B13] Hall M Frank E Holmes G Pfahringer B Reutemann P Witten IH The WEKA data mining software: an update SIGKDD Explor Newsl 2009 11 1 10 18 10.1145/1656274.1656278

[B14] Buono P Costabile M Hemmje M, Niederée C, Risse T Visualizing Association Rules in a Framework for Visual Data Mining From Integrated Publication and Information Systems to Information and Knowledge Environments, vol. 3379 2005 Berlin: Springer Berlin Heidelberg 221 231

[B15] Bruzzese D Davino C Simeon JS, Michael HB, hlen, Arturas M Visual Mining of Association Rules Visual Data Mining 2008 Berlin: Springer-Verlag 103 122

[B16] Hahsler M Chelluboina S Visualizing Association Rules in Hierarchical Groups42nd Symposium on the Interface: Statistical, Machine Learning, and Visualization Algorithms 2011 Cary, North Carolina: The Interface Foundation of North America

[B17] Krzywinski M Schein J Birol I Connors J Gascoyne R Horsman D Jones SJ Marra MA Circos: an information aesthetic for comparative genomics Genome Res 2009 19 9 1639 1645 10.1101/gr.092759.109 19541911 PMC2752132

[B18] Rainsford C Roddick J Leung K, Chan L-W, Meng H Visualisation of Temporal Interval Association Rules Intelligent Data Engineering and Automated Learning — IDEAL 2000 Data Mining, Financial Engineering, and Intelligent Agents, vol. 1983 2000 Berlin: Springer Berlin Heidelberg 91 96

[B19] Ciruvis - Circular Rule Visualization http://bioinf.icm.uu.se/~ciruvis

[B20] Bornelöv S Enroth S Komorowski J Watada J, Watanabe T, Phillips-Wren G, Howlett RJ, Jain LC Visualization of Rules in Rule-Based Classifiers Intelligent Decision Technologies, vol. 15 2012 Berlin: Springer Berlin Heidelberg 329 338

[B21] De Ruysscher D Severin D Barnes E Baumann M Bristow R Grégoire V Hölscher T Veninga T Polański A Veen E B First report on the patient database for the identification of the genetic pathways involved in patients over-reacting to radiotherapy: GENEPI-II Radiother Oncol 2010 97 1 36 39 10.1016/j.radonc.2010.03.012 20338650

[B22] Kelley Pace R Barry R Sparse spatial autoregressions Stat Probability Letters 1997 33 3 291 297 10.1016/S0167-7152(96)00140-X

[B23] Regression DataSets http://www.dcc.fc.up.pt/~ltorgo/Regression/DataSets.html

[B24] Sorokina D Caruana R Riedewald M Fink D Detecting Statistical Interactions With Additive Groves of Trees Proceedings of the 25th International Conference on Machine Learning; Helsinki, Finland. 1390282 2008 New york: ACM 1000 1007

[B25] Bornelov S Saaf A Melen E Bergstrom A Torabi Moghadam B Pulkkinen V Acevedo N Orsmark Pietras C Ege M Braun-Fahrlander C Riedler J Doekes G Kabesch M van Hage M Kere J Scheynius A Soderhall C Pershagen G Komorowski J Rule-based models of the interplay between genetic and environmental factors in childhood allergy PLoS One 2013 8 11 e80080 10.1371/journal.pone.0080080 24260339 PMC3833974

[B26] Golub TR Slonim DK Tamayo P Huard C Gaasenbeek M Mesirov JP Coller H Loh ML Downing JR Caligiuri MA Bloomfield CD Lander ES Molecular classification of cancer: class discovery and class prediction by gene expression monitoring Science 1999 286 5439 531 537 10.1126/science.286.5439.531 10521349

[B27] Alizadeh AA Eisen MB Davis RE Ma C Lossos IS Rosenwald A Boldrick JC Sabet H Tran T Yu X Powell JI Yang L Marti GE Moore T Hudson J Jr Lu L Lewis DB Tibshirani R Sherlock G Chan WC Greiner TC Weisenburger DD Armitage JO Warnke R Levy R Wilson W Grever MR Byrd JC Botstein D Brown PO Distinct types of diffuse large B-cell lymphoma identified by gene expression profiling Nature 2000 403 6769 503 511 10.1038/35000501 10676951

[B28] Draminski M Rada-Iglesias A Enroth S Wadelius C Koronacki J Komorowski J Monte Carlo feature selection for supervised classification Bioinformatics 2008 24 1 110 117 10.1093/bioinformatics/btm486 18048398

[B29] Zhang B Kirov S Snoddy J WebGestalt: an integrated system for exploring gene sets in various biological contexts Nucleic Acids Res 2005 33 Web Server issue W741 748 15980575 10.1093/nar/gki475 PMC1160236

[B30] Hvidsten TR Wilczynski B Kryshtafovych A Tiuryn J Komorowski J Fidelis K Discovering regulatory binding-site modules using rule-based learning Genome Res 2005 15 6 856 866 10.1101/gr.3760605 15930496 PMC1142476

[B31] Ciruvis - Results from the paper http://bioinf.icm.uu.se/~ciruvis/paper

