# Supplementary material for: Ciruvis: a web-based tool for rule networks and interaction detection using rule-based classifiers
Source: BMC Bioinformatics. 2014 May 12;15:139. doi: 10.1186/1471-2105-15-139 (PMC4030460; doi:10.1186/1471-2105-15-139)
Supplement: Additional file 1: Table S1 — Description of parameters and possible values for the rule submission form. [file 1471-2105-15-139-S1.pdf]

## Table S1

Description of parameters and possible values for the rule submission form.

| Parameter | Value | Description |
|---|---|---|
| Rule file | File | Local path to the rule file. |
| Threshold | Integer (0-99) | If checked, connections below the percentile threshold value are not shown in the circle. |
| Rule format | | The format of the rule file. |
| E-mail | String | A link to the results will be sent to this e-mail when the job is finished. |
| Minimum accuracy | Integer (0-99) | If checked, rules with accuracy below the threshold are not considered. |
| Minimum support | Integer (>=0) | If checked, rules with support below the threshold are not considered. |
| Groups | File | Local path to a group file. Used to defined nodes with similar color. |
| Colors | File | Local path to a color file. Used to defined colors of the nodes. |
