# Supplementary figures and images for: Ciruvis: a web-based tool for rule networks and interaction detection using rule-based classifiers
Source: BMC Bioinformatics. 2014 May 12;15:139. doi: 10.1186/1471-2105-15-139 (PMC4030460; doi:10.1186/1471-2105-15-139)

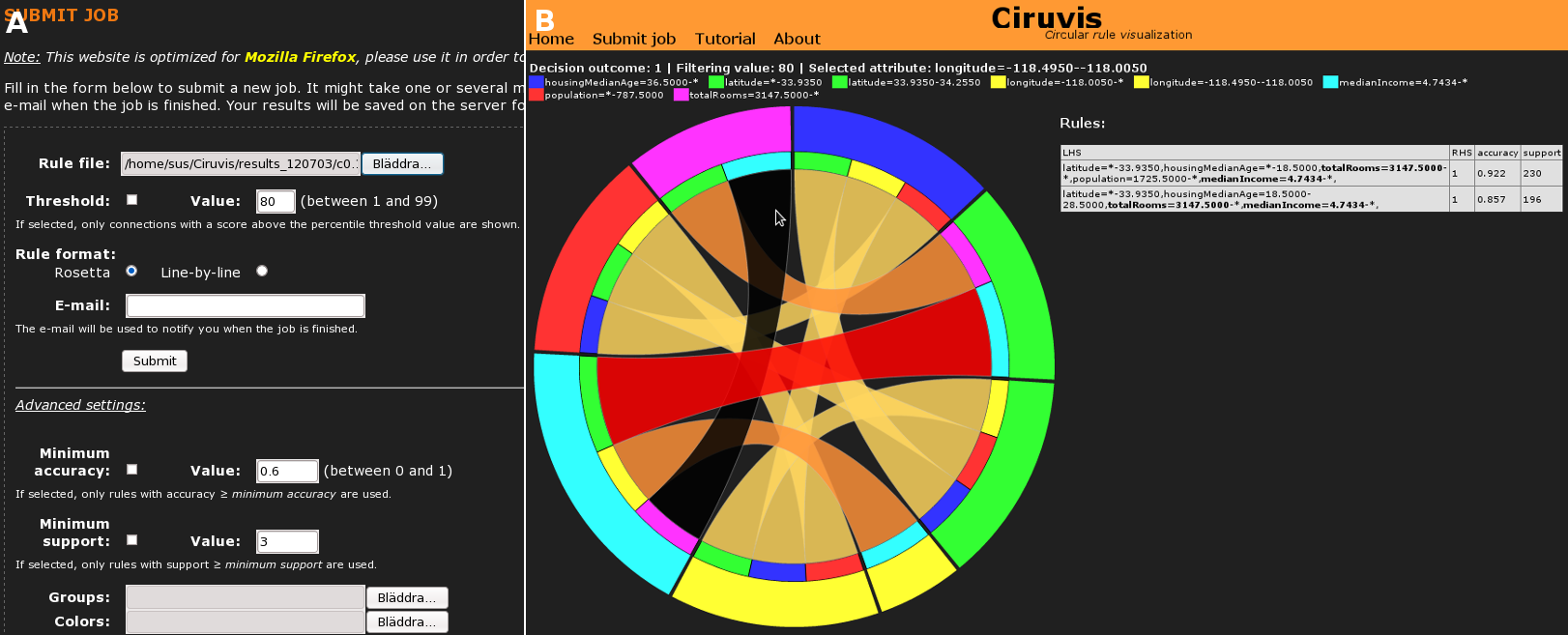

Supplement: Additional file 2: Figure S1 — (A) Ciruvis submission form. (B) Ciruvis figure for the selected outcome “1” (high). Rules for the selected connection between totalRooms = [3148,*) and medianIncome = [4.7435,*) are shown to the right. [file 1471-2105-15-139-S2.png]

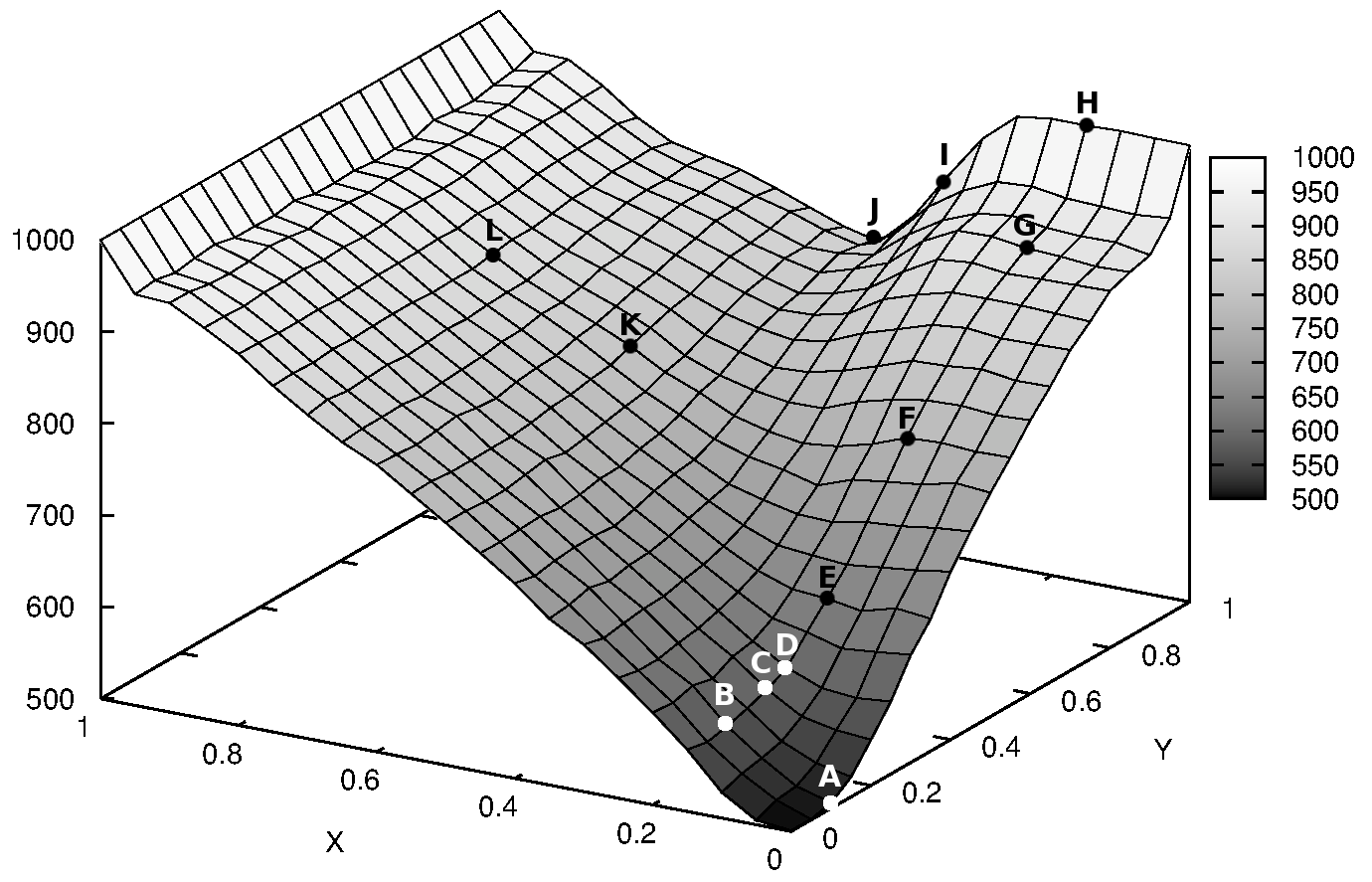

Supplement: Additional file 4: Figure S2 — The number of correctly classified objects varied for different maximal correlation (X) and level of interaction (Y). The points A-L here represent the different parameters choices in Figure 1. The average standard error of the number of correctly classified objects in the replicates with the same X and Y was 12.2 (95% CI 0.0-22.5), with datasets with the lowest X and Y having the highest variation. [file 1471-2105-15-139-S4.png]

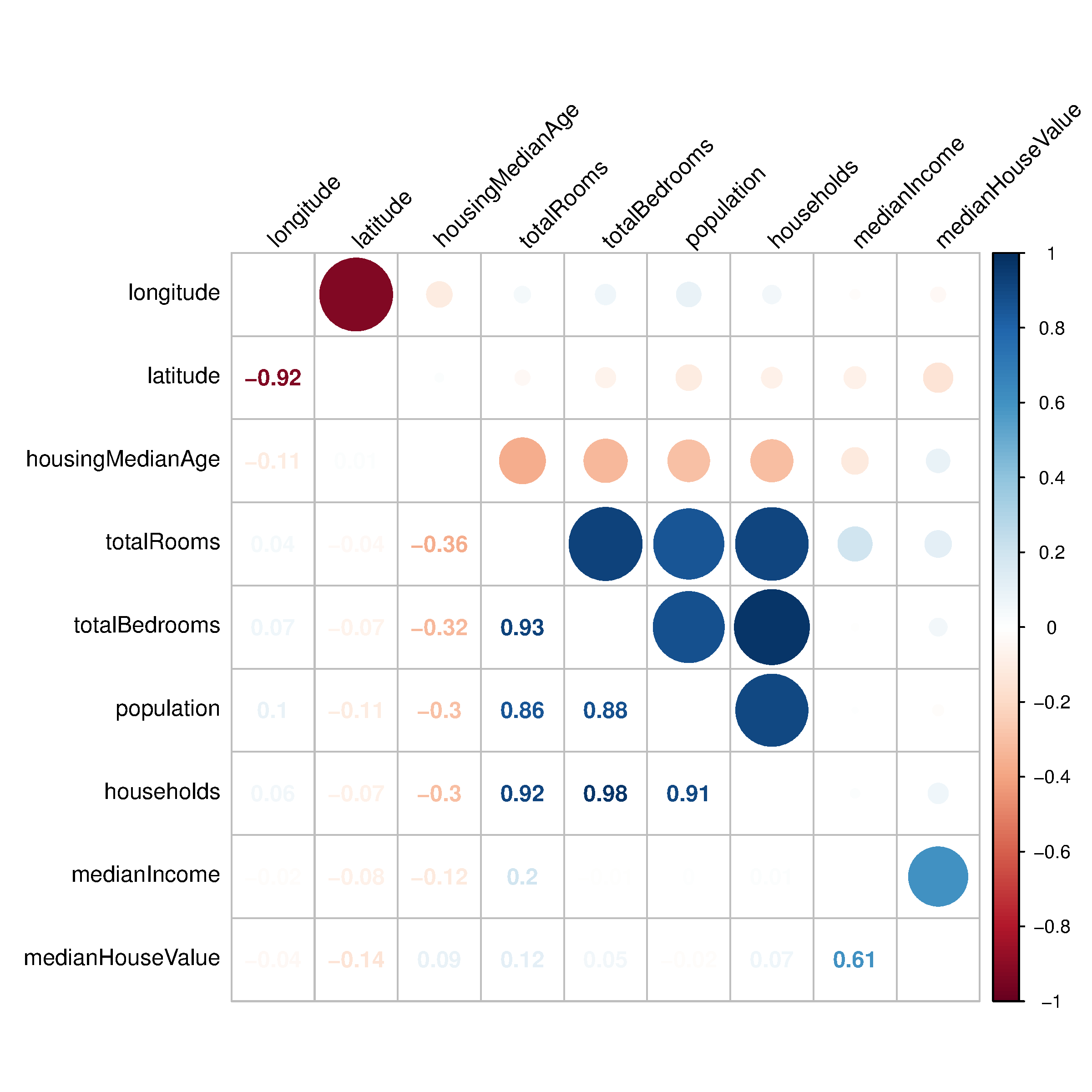

Supplement: Additional file 5: Figure S3 — Correlation between pairs of features and decision in the California Housing dataset are displayed in the upper half as filled circles with size relative to the correlation and in the lower half as values. Positive correlations are colored from white to blue (highest) and negative correlations from white and red (highest). [file 1471-2105-15-139-S5.png]

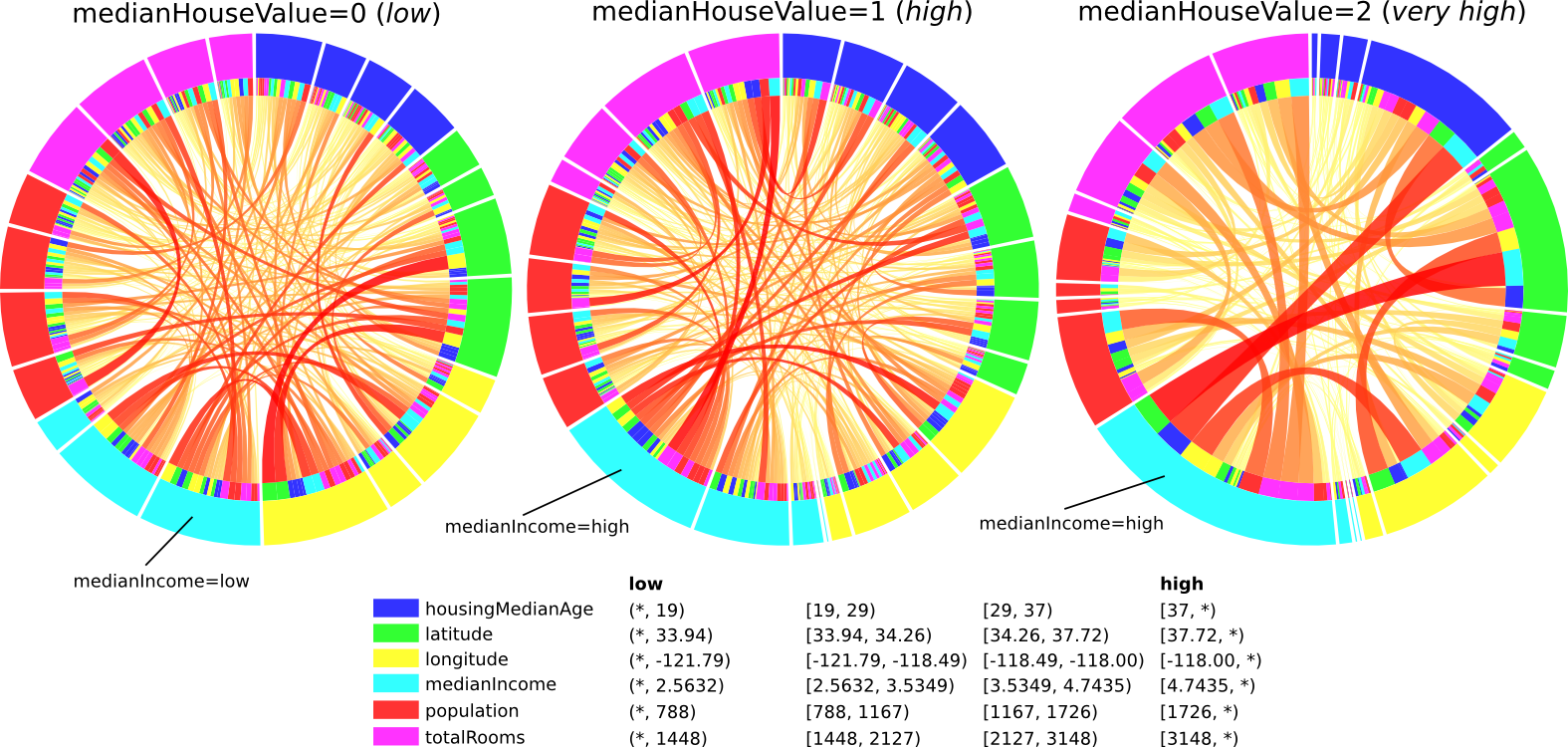

Supplement: Additional file 6: Figure S4 — Rule networks for the California housing data including the medianIncome feature. The color of the nodes shows which feature it is, and the condition values are shown in increasing order (low, middle-low, middle-high, high) on the circle. [file 1471-2105-15-139-S6.png]
