# Supplementary material for: Ciruvis: a web-based tool for rule networks and interaction detection using rule-based classifiers
Source: BMC Bioinformatics. 2014 May 12;15:139. doi: 10.1186/1471-2105-15-139 (PMC4030460; doi:10.1186/1471-2105-15-139)
Supplement: Additional file 3 — Supplementary methods. Supplementary description of the methods. [file 1471-2105-15-139-S3.pdf]

Additional file 1: Supplementary Methods

ROSETTA algorithms and parameters

The rule-based classification was done using the ROSETTA software. The parameters/algorithms for each classification problem are described below.

Simulated Data

There were 50 replicates done for each set of parameters used to generate the data. The rules were generated using the *JohnsonReducer* algorithm with approximate reducts. Rules with support ≤ 2 or accuracy ≤ 0.6 were removed during a rule filtering step.

Classifier accuracy was estimated using 10-fold cross validation for which the average results are presented (Additional file 4: Figure S2).

California Housing Data

The numerical features were discretized using the *EqualFrequencyBinning* algorithm with 3 cuts. The rules were trained using the *JohnsonReducer* algorithm with approximate reducts.

Classifier accuracy was estimated using 10-fold cross validation.

Leukemia and Lymphoma

As the data set had quite few objects after feature selection, the numerical features were discretized using the slightly more sophisticated *EntropyScaler* algorithm. The rules were trained using the *GeneticReducer* algorithm with approximate reducts.

Classifier accuracy was estimated using leave-one-out cross validation. The rules used for the rule visualization were taken from the cross validation iterations, and merged together into one rule set. The discretization cuts were determined outside of the cross validation loop, in order to guarantee that the same intervals would be used in each iteration. This may slightly overestimate the cross validation accuracy, but should benefit the rule visualization which we deemed more important.

Interaction detection in California housing data

The ten strongest connection for each of the rule networks were identified. For each of them, we calculated to relative risk (RR) and its 95 % confidence interval (CI) following [1] as

$$RR = \frac{a/(a+b)}{c/(c+d)}$$

and

$$SE(\ln RR) = \sqrt{\frac{1}{a} + \frac{1}{a+b} + \frac{1}{c} + \frac{1}{c+d}}$$

With *a* = matching the left-hand-side (LHS) of rule and the predicted outcome, *b* = matching LHS of rule and not the predicted outcome, *c* = not matching the LHS of rule and the predicted outcome, and *d* = not matching the LHS of rule and not the predicted outcome.

As a comparison for this value we calculated the RR related to each of the two conditions separately, and multiplied those together. This would imply a multiplicative model, and an interaction effect was considered to be present if the RR of the combination was significantly higher than the expected RR from the product of the two conditions.

For these rules we used a strategy similar to the RR to calculate the expected accuracy of the connection from the individual effects, see [2]. For simplicity, the results presented in the paper are the estimated accuracy compared to the observed accuracy.

Feature selection for leukemia and lymphoma

The feature selection was done using Monte Carlo feature selection implemented in the tool dmLab [3]. The number of features in the subsets, *m*, was set to the $\log_2$ of the total number of features, *d*. The number of subsets, *s*, was chosen so that each pair of features should appear together in the same subset in average ten times, which gave the formula

$$s = 10\ \frac{d(d-1)}{\log_2(d)(\log_2(d)-1)}.$$

Using this requirement, we used *s*=1,227,625 for lymphoma and *s*=3,257,405 for leukemia. The weighting parameters were set to *u*=0 and *v*=1.

The scores were assumed to follow a normal distribution, and the p-values were determined by randomization test using 100 permutations and defined as the probability that the real score (relative importance) came from the distribution obtained during the permutations. Correction for multiple testing was done using Bonferroni correction and features with $p<0.05$ after correction were considered to be significant.

References

1. Bewick V, Cheek L, Ball J: **Statistics review 11: assessing risk**. *Crit Care* 2004, **8**(4):287-291.
2. Enroth S, Bornelöv S, Wadelius C, Komorowski J: **Combinations of Histone Modifications Mark Exon Inclusion Levels**. *PLoS ONE* 2012, **7**(1):e29911.
3. Draminski M, Rada-Iglesias A, Enroth S, Wadelius C, Koronacki J, Komorowski J: **Monte Carlo feature selection for supervised classification**. *Bioinformatics* 2008, **24**(1):110-117.
