# Supplementary material for: Ciruvis: a web-based tool for rule networks and interaction detection using rule-based classifiers
Source: BMC Bioinformatics. 2014 May 12;15:139. doi: 10.1186/1471-2105-15-139 (PMC4030460; doi:10.1186/1471-2105-15-139)
Supplement: Additional file 7: Table S2 — Calculation of relative risks (RR) and their confidence intervals (CI) for each of the ten strongest connections for each outcome, as well as the expected (exp) values. Connections that had a RR significantly greater than what would be expected assuming independent effects are marked with yellow background and may indicate interaction effects. An asterisk ‘*’ in the intervals denotes + or - ∞. [file 1471-2105-15-139-S7.pdf]

Table S2

Calculation of relative risks (RR) and their confidence intervals (CI) for each of the ten strongest connections for each outcome, as well as the expected (exp) values. Connections that had a RR significantly greater than what would be expected assuming independent effects are marked with yellow background and may indicate interaction effects. An asterisk '*' in the intervals denotes + or - ∞.

Rule (LHS)	Score	RR	CI RR	RR exp	CI RR exp	Acc exp	Supp exp	Acc	Supp
**medianHouseValue=low**									
latitude=[37.71, *),longitude=[-121.79, -118.49)	2181	1.94	1.89-1.99	2.48	2.39-2.58	0.80	1448	0.84	2021
population=(*, 787),totalRooms=(*, 1447)	1229	1.15	1.11-1.19	1.38	1.33-1.45	0.60	1295	0.53	3711
population=[1166, 1725),totalRooms=[1447, 2126)	996	1.46	1.40-1.52	1.12	1.07-1.17	0.52	1291	0.68	1105
population=[1725, *),totalRooms=[2126, 3147)	985	1.34	1.27-1.41	0.89	0.85-0.94	0.44	1289	0.63	961
latitude=[33.93, 34.25),longitude=[-118.00, *)	965	1.55	1.50-1.61	0.98	0.94-1.03	0.47	1263	0.71	1351
longitude=[-121.79, -118.49),population=(*, 787)	943	1.61	1.55-1.66	1.76	1.68-1.83	0.70	1297	0.73	1463
longitude=[-121.79, -118.49),totalRooms=(*, 1447)	926	1.73	1.68-1.79	2.33	2.24-2.43	0.78	1419	0.79	1301
latitude=[34.25, 37.71),longitude=[-121.79, -118.49)	913	1.57	1.52-1.62	1.67	1.60-1.74	0.68	1278	0.70	2649
longitude=[-121.79, -118.49),population=[787, 1166)	911	1.57	1.51-1.63	1.63	1.56-1.70	0.68	1268	0.72	1246
longitude=[-118.00, *),totalRooms=[2126, 3147)	869	1.05	0.99-1.11	1.01	0.96-1.06	0.48	1276	0.50	1302
**medianHouseValue=high**									
population=[1725, *),totalRooms=[3147, *)	2307	1.23	1.19-1.27	1.35	1.29-1.41	0.59	1299	0.56	3734[
population=[787, 1166),totalRooms=[1447, 2126)	1206	1.06	1.01-1.10	0.96	0.91-1.00	0.46	1288	0.50	2417
housingMedianAge=(*, 18),totalRooms=[3147, *)	1090	1.10	1.06-1.15	1.18	1.13-1.23	0.54	1318	0.52	2360
population=(*, 787),totalRooms=(*, 1447)	1062	0.86	0.83-0.90	0.67	0.63-0.70	0.34	1308	0.42	3711
longitude=(*, -121.795),totalRooms=[3147, *)	1025	1.56	1.50-1.62	1.81	1.73-1.88	0.69	1356	0.72	1310
population=[1166, 1725),totalRooms=[2126, 3147)	977	1.12	1.07-1.16	1.11	1.06-1.16	0.51	1290	0.53	2380
longitude=(*, -121.795),population=(*, 787)	902	1.13	1.07-1.18	1.29	1.23-1.35	0.58	1270	0.53	1506
population=[787, 1166),totalRooms=[2126, 3147)	890	1.28	1.22-1.33	1.15	1.10-1.20	0.53	1293	0.59	1673
latitude=(*, -33.93),population=[787, 1166)	835	1.30	1.24-1.36	1.29	1.23-1.34	0.57	1303	0.61	1272
latitude=(*, -33.93),totalRooms=[1447, 2126)	775	1.15	1.09-1.21	1.15	1.10-1.20	0.53	1286	0.54	1268
**medianHouseValue=very high**									
population=[1166, 1725),totalRooms=[3147, *)	54	3.12	2.67-3.64	1.13	0.93-1.38	0.05	1287	0.13	1275
longitude=[-118.49, -118.00),totalRooms=[3147, *)	43	3.08	2.58-3.68	2.56	2.14-3.07	0.09	1304	0.14	883
latitude=[33.93, 34.25),totalRooms=[3147, *)	40	2.51	2.11-2.99	2.52	2.11-3.02	0.09	1290	0.11	1169
latitude=[33.93, 34.25),longitude=[-118.49, -118.00)	40	2.06	1.80-2.35	3.08	2.58-3.68	0.10	1299	0.08	3287
population=(*, 787),totalRooms=[2126, 3147)	38	5.10	3.79-6.85	2.05	1.71-2.47	0.08	1294	0.24	147
housingMedianAge=[36, *),latitude=[33.93, 34.25)	38	1.99	1.70c-2.32	3.34	2.80-3.98	0.11	1378	0.09	1996
longitude=[-118.49, -118.00),population=[1166, 1725)	35	1.27	1.02-1.59	1.38	1.14-1.68	0.06	1289	0.06	1333
latitude=[33.93, 34.25),population=[1166, 1725)	35	1.29	1.04-1.61	1.36	1.12-1.66	0.06	1275	0.06	1312
housingMedianAge=[36, *),totalRooms=[3147, *)	32	4.44	3.62-5.44	2.77	2.32-3.32	0.10	1382	0.20	411
population=[787, 1166),totalRooms=[3147, *)	30	5.52	4.01-7.60	1.73	1.44-2.09	0.07	1293	0.26	112

'Rule (LHS)' is the left-hand-side (condition) part of the rule, 'Score' is the Ciruvis connection score, 'RR' is the relative risk, 'RR CI' is the 95 % confidence interval of the RR, 'RR exp' and 'RR CI exp' is the expected RR and CI assuming multiplicative effects. 'Acc' is the rule accuracy, 'Supp' is the rule support. 'Acc exp' and 'Supp exp' is the expected accuracy and support assuming independent effects.
