# Supplementary material for: Ciruvis: a web-based tool for rule networks and interaction detection using rule-based classifiers
Source: BMC Bioinformatics. 2014 May 12;15:139. doi: 10.1186/1471-2105-15-139 (PMC4030460; doi:10.1186/1471-2105-15-139)
Supplement: Additional file 8: Table S3 — The 30 most significant features for the lymphoma data (p-values calculated by MCFS). The original name refer to the internal name in the source data set. The gene name is given whenever it was available. The range for the discretized expression values are given as Low and High. [file 1471-2105-15-139-S8.pdf]

Table S3

The 30 most significant features for the lymphoma data (p-values calculated by MCFS). The original name refer to the internal name in the source data set. The gene name is given whenever it was available. The range for the discretized expression values are given as Low and High.

Original name	Gene	Low	High	p-value
GENE2368X	*PRKCB*	(*, 1.14)	[1.14, *)	<2.7E-297
GENE508X	*RAD23B*	(*, -0.70)	[-0.70, *)	1.2E-241
GENE1672X	*GPX1*	(*, -0.89)	[-0.89, *)	4.2E-199
GENE586X	*PPP2R1A*	(*, -0.76)	[-0.76, *)	9.4E-188
GENE1647X	*CTSB*	(*, 0.02)	[0.02, *)	5.5E-163
GENE669X		(*, -1.04)	[-1.04, *)	2.5E-158
GENE528X		(*, -0.82)	[-0.82, *)	2.2E-150
GENE1662X		(*, -0.40)	[-0.40, *)	3.4E-149
GENE1625X		(*, -0.20)	[-0.20, *)	1.6E-138
GENE651X	*TUBB*	(*, -1.27)	[-1.27, *)	4.0E-137
GENE2374X	*PRKCB'*	(*, 1.62)	[1.62, *)	3.3E-134
GENE639X	*HDGF*	(*, -1.20)	[-1.20, *)	8.4E-130
GENE529X		(*, -0.72)	[-0.72, *)	3.5E-127
GENE1661X	*AIF1*	(*, -0.57)	[-0.57, *)	9.2E-127
GENE2166X		(*, 0.50)	[0.50, *)	1.4E-124
GENE459X	*PPEF1*	(*, -0.38)	[-0.38, *)	4.7E-124
GENE647X	*MIF*	(*, -0.48)	[-0.48, *)	1.7E-123
GENE631X	*ADSL*	(*, -0.25)	[-0.25, *)	1.9E-122
GENE2391X		(*, 1.23)	[1.23, *)	1.5E-121
GENE2402X		(*, 1.28)	[1.28, *)	8.2E-109
GENE834X	*CALR*	(*, -0.40)	[-0.40, *)	2.5E-105
GENE2668X	*MXI1*	(*, 0.52)	[0.52, *)	1.6E-103
GENE1610X	*CXCL9*	(*, -0.69)	[-0.69, *)	3.0E-103
GENE1622X	*CD63*	(*, -0.75)	[-0.75, *)	5.0E-103
GENE1602X	*PCBP2*	(*, -0.39)	[-0.39, *)	2.3E-102
GENE786X	*RPS2*	(*, -0.73)	[-0.73, *)	2.2E-101
GENE616X	*SUMO1*	(*, -0.17)	[-0.17, *)	1.6E-100
GENE622X	*NT5C2*	(*, -0.20)	[-0.20, *)	4.1E-100
GENE1599X	*SULT1A1*	(*, -0.68)	[-0.68, *)	1.2E-099
GENE1731X	*MYB*	(*, -0.59)	[-0.59, *)	4.3E-098
