# Supplementary material for: Ciruvis: a web-based tool for rule networks and interaction detection using rule-based classifiers
Source: BMC Bioinformatics. 2014 May 12;15:139. doi: 10.1186/1471-2105-15-139 (PMC4030460; doi:10.1186/1471-2105-15-139)
Supplement: Additional file 9: Table S4 — The 30 most significant features for the leukemia data (p-values calculated by MCFS). The original name refer to the internal name in the source data set. The gene name is given whenever it was available. The range for the discretized expression values are given as Low, Medium (if applicable) and High. [file 1471-2105-15-139-S9.pdf]

Table S4

The 30 most significant features for the leukemia data (p-values calculated by MCFS). The original name refer to the internal name in the source data set. The gene name is given whenever it was available. The range for the discretized expression values are given as Low, Medium (if applicable) and High.

Original name	Gene	Low	Medium	High	p-value
X95735_at	*ZYX*	(*, 994)		[994, *)	<2.7E-297
M27891_at	*CST3*	(*, 947)		[947, *)	<2.7E-297
M77142_at	*TIA1*	(*, 81)		[81, *)	<2.7E-297
U46499_at	*MGST3*	(*, 157)		[157, *)	<2.7E-297
X70297_at	*CHRFAM7A*	(*, 339)		[339, *)	<2.7E-297
D88422_at	*CSTA*	(*, 235)		[235, *)	2.7E-297
M55150_at	*FAH*	(*, 1346)		[1346, *)	8.3E-282
U22376_cds2_s_at	*MYB*	(*, 1423)		[1423, *)	6.6E-265
L09209_s_at	*APLP2*	(*, 993)		[993, *)	6.1E-256
D14874_at	*ADM*	(*, 185)		[185, *)	4.8E-236
M31523_at	*TCF3*	(*, 416)		[416, *)	9.4E-219
J05243_at	*SPTAN1*	(*, 136)		[136, *)	1.3E-213
M91432_at	*ACADM*	(*, 394)		[394, *)	1.9E-206
M31166_at	*PTX3*	(*, 84)		[84, *)	1.2E-188
M23197_at	*CD33*	(*, 313)		[313, *)	4.5E-186
U50136_rna1_at	*LTC4S*	(*, 1341)		[1341, *)	2.1E-185
M16038_at	*LYN*	(*, 652)		[652, *)	6.6E-178
M92287_at	*CCND3*	(*, 1870)		[1870, *)	2.1E-176
M12959_s_at	*TRA*	(*, 821)		[821, *)	4.3E-173
M81933_at	*CDC25A*	(*, 128)		[128, *)	1.8E-164
U73737_at	*MSH6*	(*, 93)	[93, 165)	[165, *)	9.9E-159
U09087_s_at	*TMPO*	(*, 78)		[78, *)	6.2E-147
X15949_at	*IRF2*	(*, 30)	[30, 85)	[85, *)	5.1E-145
X74262_at	*RBBP4*	(*, 626)		[626, *)	4.5E-136
U02020_at	*NAMPT*	(*, 382)		[382, *)	2.1E-134
Y12670_at	*LEPR*	(*, 642)		[642, *)	2.7E-123
X17042_at	*SRGN*	(*, 5551)		[5551, *)	6.9E-120
M83652_s_at	*CFP*	(*, 542)		[542, *)	4.1E-117
X59417_at	*PSMA6*	(*, 1797)		[1797, *)	1.3E-116
Y00787_s_at	*IL8*	(*, 1687)		[1687, *)	1.8E-116
